# Antitumor Efficacy of the Herbal Recipe Benja Amarit against Highly Invasive Cholangiocarcinoma by Inducing Apoptosis both In Vitro and In Vivo

**DOI:** 10.3390/ijms21165669

**Published:** 2020-08-07

**Authors:** Rittibet Yapasert, Nirush Lertprasertsuk, Subhawat Subhawa, Juthathip Poofery, Bungorn Sripanidkulchai, Ratana Banjerdpongchai

**Affiliations:** 1 Department of Biochemistry, Faculty of Medicine, Chiang Mai University, Chiang Mai 50200, Thailand; rittibet_ya@cmu.ac.th (R.Y.); subhawat_s@cmu.ac.th (S.S.); juthathip_p@cmu.ac.th (J.P.); 2 Department of Pathology, Faculty of Medicine, Chiang Mai University, Chiang Mai 50200, Thailand; nlertpra@hotmail.com; 3 Center for Research and Development of Herbal Health Products (CRD-HHP), Faculty of Pharmaceutical Sciences, Khon Kaen University, Khon Kaen 40002, Thailand; bungorn@kku.ac.th; 4 Department of Pharmaceutical Chemistry, Faculty of Pharmaceutical Sciences, Khon Kaen University, Khon Kaen 40002, Thailand

**Keywords:** Benja Amarit, BJA, herbal recipe, cholangiocarcinoma, liver cancer, apoptosis, autophagy, in vitro, in vivo

## Abstract

Thailand is the country with highest incidence and prevalence of cholangiocarcinoma (CCA) in the world. Due to the frequently late diagnosis that is associated with this disease, most CCA patients are prescribed chemotherapy as a form of treatment. However, CCA is able to resist the presently available chemotherapy, so to the prognosis of this disease is still very poor. In this study, we investigated the anticancer potential of a Thai herbal recipe, Benja Amarit (BJA) against CCA and the relevant mechanisms of action that are involved. We found that BJA inhibited CCA cell viability in a dose-dependent manner, especially in highly invasive KKU-213 cells. The extract induced mitochondrial- and caspase-dependent apoptosis in CCA cells by regulating the nuclear factor-κB (NF-κB) signaling pathway. BJA also triggered autophagy in CCA cells. Nonetheless, the inhibition of autophagy enhanced BJA-induced CCA cell death via apoptosis. An in vivo xenograft model revealed the growth-inhibiting and death-inducing effects of BJA against CCA by targeting apoptosis. However, general toxicity to blood cells, kidneys and the liver, as well as changes in body weight, did not appear. Our findings suggest that the herbal recipe BJA might be used as a potentially new and effective treatment for cholangiocarcinoma patients.

## 1. Introduction

Liver cancer, or primary hepatic cancer, consists of two main histological types, hepatocellular carcinoma (HCC) and cholangiocarcinoma (CCA). HCC is the most common type of liver cancer worldwide [1]. In Thailand, CCA is highly prevalent and is the predominant pathological type, accounting for more than 80% of all detected primary liver cancer [2,3]. Due to its aggressiveness and the poor prognosis often given to patients suffering from this disease, CCA is still associated with high mortality rates, especially in the northeast of Thailand [1,4,5]. At present, only a quarter of CCA patients are eligible for surgical resection of the tumor, while most of them are prescribed chemotherapy as a form of treatment [6]. Unfortunately, CCA is able to resist conventional chemotherapy by escaping with several tolerance mechanisms [7,8]. Hence, it is vitally important to develop a novel medication with high effectiveness for CCA treatment [9,10].

Apoptosis, or type I programmed cell death, plays an important role in cellular homeostasis and in protecting organisms against cancer cell development [11,12]. Apoptosis is also known as a tumor suppressor pathway; therefore, the upregulation of apoptosis within cancer cells is recognized as an effective therapeutic strategy [13,14]. Meanwhile, the autophagy response to therapy does not always lead to cell death. It can play a protective role against the treatment, which depends upon the biology and context of each specific cancer cell [15,16]. Thus, the determination of an autophagic response to each therapeutic drug for each cancer cell type is important for identifying signs of drug responsiveness and/or resistance [17,18].

Benja Amarit (BJA) is a traditional Thai herbal recipe, which has been used in the treatment of liver cancer for more than a century in Thailand. The relevant bioactive phytochemical compounds, which include piperine, gambogic acid and xanthotoxol, have been identified in our previous study [19]. An in vitro study illustrated its anticancer potential for colon cancer and, in particular, hepatocellular carcinoma by inducing both extrinsic and intrinsic apoptosis pathways through reactive oxygen species production and endoplasmic reticulum (ER) stress. The extract also displays growth-inhibiting effects on hepatocellular carcinoma in vivo [19,20]. Moreover, the safety and potential effectiveness of BJA in patients with hepatocellular carcinoma have also been examined [21]. However, the anticancer activity and the mechanisms of action of BJA on cholangiocarcinoma (CCA) have not yet been investigated. In this study, we found that the BJA extract could induce both apoptosis and autophagy in CCA cells. Additionally, it effectively suppressed CCA growth in nude mice.

## 2. Results

### 2.1. BJA-Inhibited CCA Cell Viability

**Figure 1:**
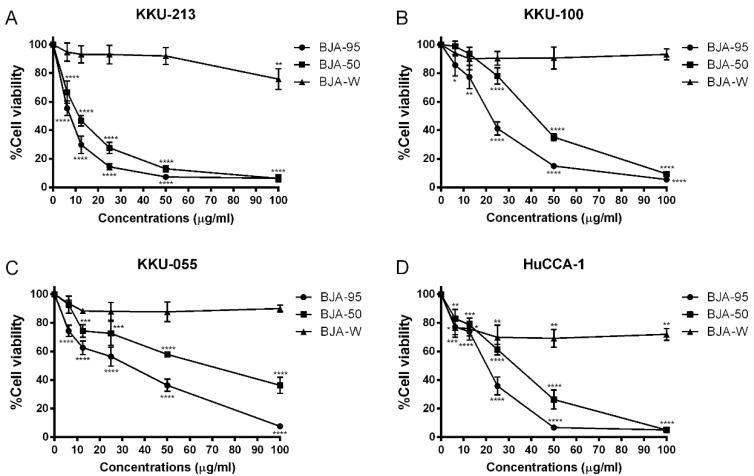
Cytotoxic effect of Benja Amarit (BJA) against cholangiocarcinoma (CCA) cell lines. Human cholangiocarcinoma (**A**) KKU-213, (**B**) KKU-100, (**C**) KKU-055 and (**D**) HuCCA-1 cells were treated with 0–100 µg/mL of BJA-95, BJA-50 and BJA-W for 24 h. Cell viability was then measured using an MTT assay. Results are shown as mean ± SD values from three repeated independent experiments. (*) *p* < 0.05, (**) *p* < 0.01, (***) *p* < 0.001 and (****) *p* < 0.0001 when compared with the control (without treatment).

To examine the cytotoxic effects of BJA on human cholangiocarcinoma (CCA) cell lines, KKU-213, KKU-100, KKU-055 and HuCCA-1 cells were treated with 95% ethanolic extract of BJA (BJA-95), 50% ethanolic extract of BJA (BJA-50) and a water extract of BJA (BJA-W) for 24 h. We found that BJA-95 and BJA-50 inhibited CCA cell viability in a dose-dependent manner. However, BJA-W was not found to be toxic in the same concentration range (Figure 1). Intriguingly, BJA-95 presented the lowest value of IC50, with significant differences from BJA-50 and BJA-W in all of the cell lines. Additionally, BJA-95 on highly invasive type KKU-213 cells revealed the least amount of IC50 when compared to the other CCA cells (Table 1). Thus, the most effective extract, BJA-95, and the most sensitive cancer cell line, KKU-213, were appropriately selected for an investigation of the underlying mechanisms.

### 2.2. BJA-Induced Apoptosis in CCA Cells

**Figure 2:**
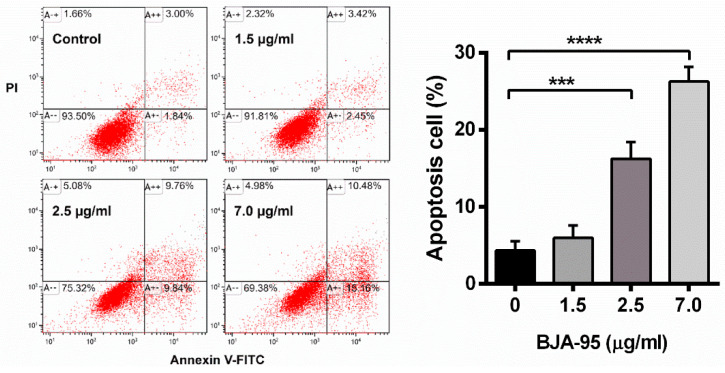
Apoptosis induction by BJA in CCA cells. KKU-213 cells were treated with indicated concentrations of BJA-95 for 24 h. Cells were stained with annexin V-fluorescein isothiocyanate and propidium iodide (annexin V-FITC/PI) and analyzed by flow cytometry. The bar graph presents the percentage of apoptotic cells. Results are shown as mean ± SD, *n* = 3. (***) *p* < 0.001 and (****) *p* < 0.0001 when compared with the control.

**Figure 3:**
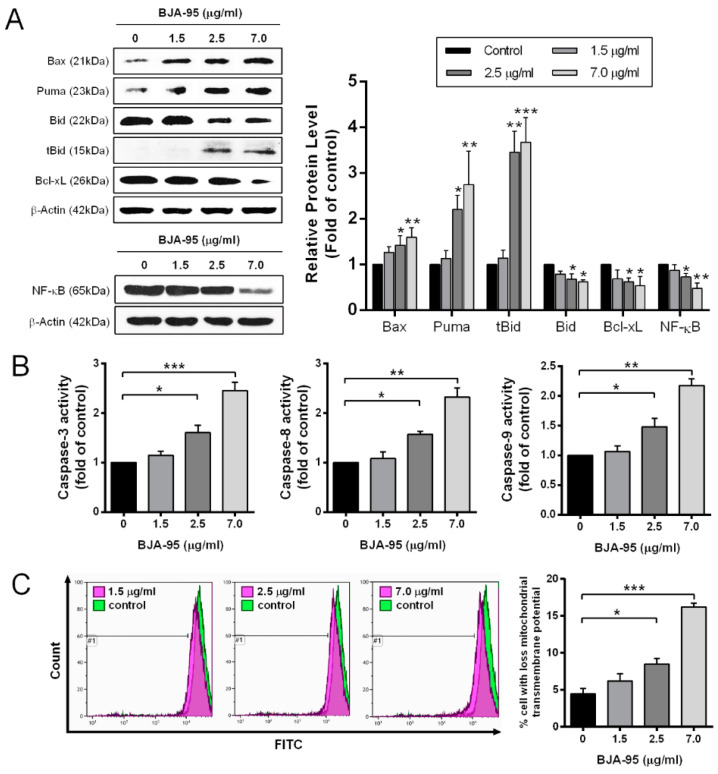
The effects of BJA on the expression of apoptotic-related proteins, caspase activities and mitochondrial transmembrane potential in CCA cells. KKU-213 cells were treated with indicated concentrations of BJA-95 for 24 h. (**A**) Expression levels of pro-apoptotic proteins (Bax, Puma and tBid) and anti-apoptotic proteins (Bcl-xL and NF-κB) in KKU-213 cells after stimulation with BJA-95 at different concentrations. The amount of proteins applied in each lane was normalized by β-actin. (**B**) Caspase-3, -8 and -9 activities at indicated concentrations were investigated and compared to the control. (**C**) KKU-213 cells were stained with DiOC_6_ and analyzed by flow cytometry to examine mitochondrial transmembrane potential. The bar graphs present the percentage of the cells with a loss of mitochondrial transmembrane potential after BJA-95 treatment for 24 h. Results are shown as mean ± SD, *n* = 3. (*) *p* < 0.05, (**) *p* < 0.01, (***) *p* < 0.001 compared with the control (without treatment).

To elucidate the mode of action of BJA-95 through apoptosis induction, KKU-213 cells were treated with BJA-95 at 10%, 20% and 50% of the inhibitory concentration for 24 h. Apoptotic cells were then quantitated by annexin V-fluorescein isothiocyanate (FITC) and propidium iodide (PI) double staining. As is shown in Figure 2, the percentage of apoptotic cells significantly increased in a dose-dependent manner. Moreover, BJA-95 significantly upregulated the expression levels of the following pro-apoptotic proteins: Bax, Puma and tBid, whereas it downregulated the apoptosis-inhibiting proteins Bcl-xL and NF-κB (Figure 3A). Caspase activity was determined in order to confirm the involvement of caspase activation in BJA-95-induced apoptosis. The results revealed that the activity of caspase-3, -8 and -9 significantly increased in a dose-dependent manner after BJA-95 treatment (Figure 3B). Additionally, depolarization of the mitochondrial transmembrane potential, resulting from mitochondrial outer membrane permeabilization (MOMP), was exhibited for the purposes of contributing to apoptosis induction [22,23]. Hence, the effect of BJA-95 on the alteration of mitochondrial transmembrane potential was examined. We found that, after treatment with BJA-95, the percentage of cells with a loss of mitochondrial transmembrane potential significantly increased, as is shown in Figure 3C. This indicated that BJA-95 induced mitochondrial and caspase-dependent apoptosis in KKU-213 cells via the NF-κB signaling pathways.

### 2.3. BJA-Triggered Autophagy in CCA Cells

**Figure 4:**
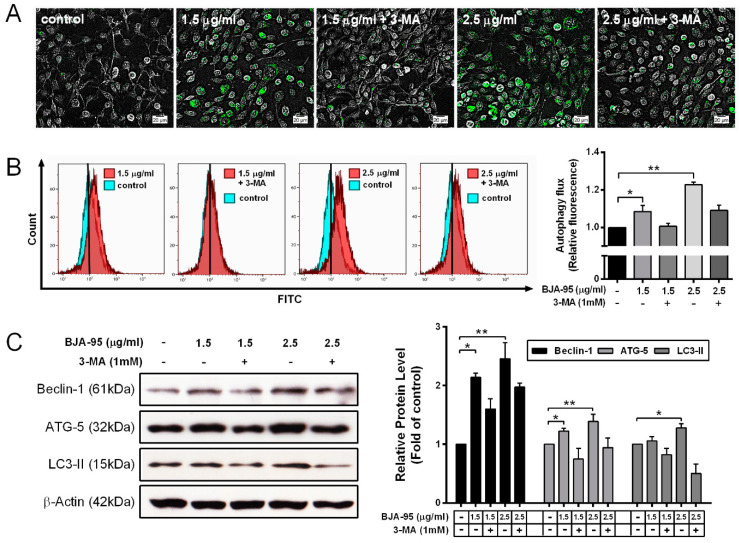
BJA-induced autophagy in CCA cells. The KKU-213 cells were treated with indicated concentrations of BJA-95. Specific fluorescence probes were then used to stain autophagic vacuoles. (**A**) Representative photos of autophagic vacuoles were examined under fluorescence microscopy. (**B**) Autophagic flux was quantitated by flow cytometry. (**C**) The expression levels of autophagy marker proteins in KKU-213 cells after stimulation with BJA-95 at different concentrations. The amount of proteins applied in each lane was normalized by β-actin. Results are shown as mean ± SD values from three repeated independent experiments. (*) *p* < 0.05 and (**) *p* < 0.01 when compared with the control.

Previous studies have shown that autophagy is considered as another response mechanism of CCA to several chemotherapy treatments [24,25,26]. Hence, in order to determine the effect of BJA-95 on the autophagy induction in CCA, autophagic vacuoles were imaged by fluorescence microscopy (Figure 4A) and were then quantitated by flow cytometry (Figure 4B). The results revealed that, after treatment with BJA-95, autophagy flux gradually increased in a dose-dependent manner. Consistent with the expression levels of autophagic marker proteins, Beclin-1, ATG-5 and LC3-II significantly increased with BJA-95 treatment, as is shown in Figure 4C. Additionally, 3-methyladenine (3-MA), an autophagy inhibitor, was used to confirm BJA-95-induced autophagy. The results indicated that 3-MA inhibited the formation of autophagic vacuoles and the expression levels of autophagic marker proteins, suggesting that BJA-95 induced autophagy in KKU-213 cells.

### 2.4. Inhibition of Autophagy-Enhanced BJA-Induced Apoptosis

**Figure 5:**
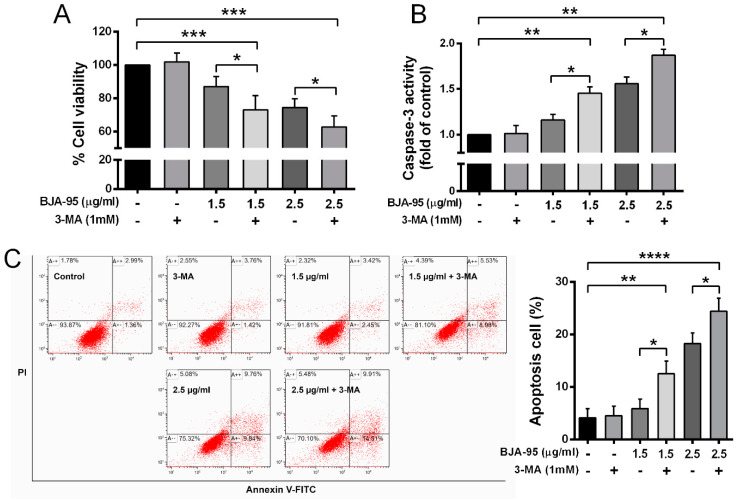
Suppression of autophagy-enhanced BJA-induced apoptosis. (**A**) KKU-213 cells were treated with BJA-95 in the presence or absence of the autophagic inhibitor 3-MA and cell viability was measured using an MTT assay. (**B**) Caspase-3 activity and (**C**) the percentage of apoptotic cells after treatment with BJA-95 in the presence or absence of an autophagic inhibitor. Results are shown as mean ± SD values from three repeated independent experiments. (*) *p* < 0.05, (**) *p* < 0.01, (***) *p* < 0.001 and (****) *p* < 0.0001 when compared with the control.

The autophagic response of cancer cells to therapeutics may play different roles in the promotion or suppression of cell death, both of which are dependent upon the individual cell biology [16,18,27]. Hence, in order to understand the role of BJA-95-induced autophagy in KKU-213 cells, the cell viability after BJA-95 treatment with or without the autophagic inhibitor 3-MA was examined. We found that the suppression of the autophagic response to BJA-95 led to significant decreases in cell viability (Figure 5A). This outcome indicated that autophagy plays a role in maintaining CCA cell survival upon BJA treatment. It was found that the inhibition of protective autophagy can sensitize cells to apoptosis in several cancer therapies [28,29,30]. Thus, we next determined the influence of autophagy inhibition on the level of apoptosis in BJA-treated cells. We found that the percentage of apoptotic cells (Figure 5C) and caspase-3 activity (Figure 5B) significantly increased when autophagy was inhibited in BJA-treated cells. This indicated that the suppression of autophagy significantly enhanced the cytotoxic effect of BJA-95 by elevating apoptosis.

### 2.5. Anti-Tumor Activity of BJA in the In Vivo Model

**Figure 6:**
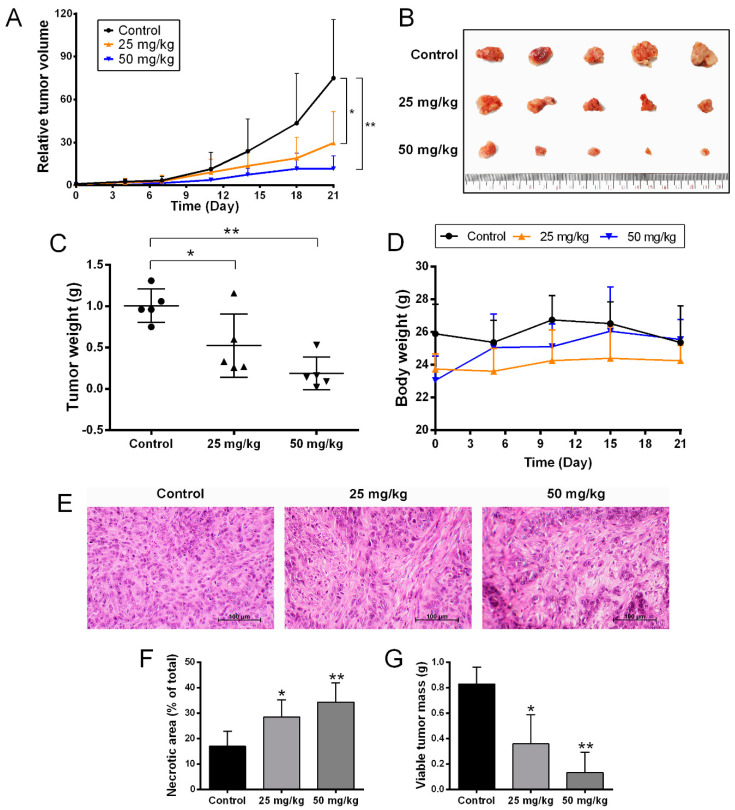
BJA-95 inhibited CCA growth in vivo. (**A**) Relative tumor volume. (**B**) Representative photos of the tumor mass. (**C**) Tumor weight of treatment groups compared to the control group. (**D**) Body weights of mice. (**E**) Hematoxylin and eosin (H&E) staining of tumor tissues. (**F**) Percentage of necrotic area in tumor tissues and (**G**) viable tumor mass. Results are shown as mean ± SD values (*n* = 5). (*) *p* < 0.05 and (**) *p* < 0.01 when compared with the control group.

**Figure 7:**
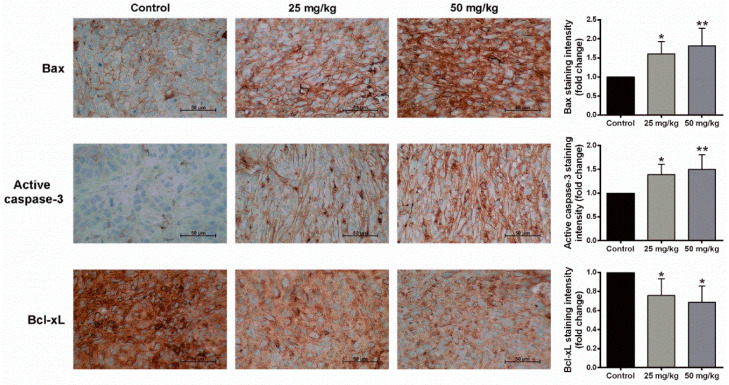
BJA-95 inhibited CCA growth by targeting apoptosis. Representative photos of the immunohistochemistry of Bax, active caspase-3 and Bcl-xL at a magnification of ×400. Bar graphs illustrate the levels of protein expression. Results are shown as mean ± SD (*n* = 5). (*) *p* < 0.05 and (**) *p* < 0.01 when compared to the control group.

Our current in vitro study revealed the cytotoxic effect of BJA on CCA cells. Hence, the most effective extract, BJA-95, along with the most sensitive CCA cell line, KKU-213, were appropriately selected for the in vivo experiment. A nude mouse xenograft model was achieved by subcutaneous inoculation of KKU-213 cells. BJA-95 was then fed to mice by oral gavage daily for 20 days at 0, 25 and 50 mg/kg. The results revealed that tumor volume (Figure 6A,B) and tumor weight (Figure 6C) in BJA-95 treatment groups were lower and significantly different from the control group. Notably, the extract did not affect general animal behavior and physical activity. The body weights of mice treated with BJA-95 were not significantly different from the control group (Figure 6D). In addition, the extract showed no discernible toxicity to animal blood cells, kidneys or the liver (Table 2 and Figure S1). Hematoxylin and eosin (H&E) staining (Figure 6E) revealed that BJA treatment increased the necrotic area of tumor tissue (Figure 6F). Moreover, approximately 80% of the viable tumor mass was reduced when compared to the control (Figure 6G). To confirm our findings on the inhibitory effects on tumor growth, the expression of apoptotic-related proteins in the tumor tissues was determined using immunohistochemical analysis. As is shown in Figure 7, BJA-95 caused the expression of the pro-apoptotic protein Bax and active caspase-3 to increase in all treatment groups, whereas anti-apoptotic Bcl-xL protein expression decreased when compared with the control. This indicated that BJA-95 inhibited CCA growth by targeting apoptosis.

## 3. Discussion

Chemotherapy is recommended for more than 70% of patients with cholangiocarcinoma (CCA) due to the frequently late diagnosis that is associated with this disease [9]. However, CCA is able to evade chemotherapy by powerful mechanisms of chemoresistance [7,8], which means that its response to available chemotherapeutic agents is very low [31,32]. In this research study, we have discovered a novel effective alternative treatment for CCA involving the herbal recipe Benja Amarit (BJA). The potential anticancer activity of BJA against CCA and the mechanisms that are involved have been demonstrated in this study. BJA-95 and BJA-50 inhibited the cell viability of CCA in a dose-dependent manner, while the water extract of BJA was found to be non-toxic to all CCA cell lines. Intriguingly, BJA-95 seemed to be the most effective extract by presenting the lowest IC50 value in all CCA cells when compared to BJA-50 and BJA-W, whereas it was found to be less toxic to normal cholangiocytes and other normal cells (Table S1). Meanwhile, highly invasive KKU-213 cells were found to be the most responsive CCA cells to BJA. Hence, the most effective extract, BJA-95, and the most sensitive cell line, KKU-213, were chosen for a subsequent study on the relevant mechanisms of action.

Targeting apoptosis is one of the most attractive strategies in cancer treatments due to the fact that the apoptotic cells are removed by phagocytes without causing inflammation and tissue damage [33]. Our study found that apoptosis was induced in KKU-213 cells after being treated with BJA-95 for 24 h. Moreover, BJA-95 increased the percentage of cells with the loss of mitochondrial transmembrane potential and the activities of caspase-3, -8 and -9, indicating that BJA-95 could induce KKU-213 cell apoptosis through the mitochondrial and caspase cascade pathways. Nuclear factor NF-κB plays an important role in countering the induction of apoptosis [34,35] by regulating the transcription of apoptotic-related genes, such as *Bcl-2* [36,37,38], thus modulating the survival and growth of various cancers, including CCA. In this study, we found that the expression level of NF-κB was reduced by BJA-95 treatment, which was consistent with the decreased expression levels of anti-apoptotic proteins and the increased expression levels of pro-apoptotic proteins. Therefore, it indicated that BJA-95 promoted apoptosis through the abrogation of the NF-κB signaling pathway.

Previous reports have shown that various plant extracts can induce both apoptosis and autophagy in CCA cells [24,39]. According to our current results, BJA could induce apoptosis in KKU-213 cells; therefore, the other mode of action via autophagy was investigated. Intriguingly, BJA-95 could induce autophagic vacuole formation and autophagic flux within KKU-213 cells, indicated by staining with a specific fluorescence probe. This outcome was consistent with the increased expression levels of autophagic marker proteins. This autophagy was confirmed by pretreatment with the autophagy inhibitor 3-methyladenine (3-MA). However, it is important to understand the role of an autophagy-response to BJA-95, as the response could lead to either cancer cell death or survival [40,41,42]. We found that the suppression of such autophagy by 3-MA could lead to significant decreases in cell viability. This would indicate that autophagy plays a protective role in the response to the BJA-95 treatment of KKU-213 cells. It is reported that the inhibition of the autophagy response to therapy can alter the apoptotic threshold [43,44], leading to an enhancement of the effects of various anticancer agents and the promotion of the chemo-sensitization of the tumors that are resistant to treatment [45,46,47,48]. Current studies have shown that a combination treatment with an autophagy inhibitor could sensitize the anticancer effect of BJA by increasing the apoptotic death of CCA cells. Therefore, the inhibition of the protective autophagy mechanism was the novel therapeutic strategy to enhance the treatment efficacy of BJA, which might then overcome subsequent therapeutic resistance in CCA.

The current in vitro study revealed the death-inducing effect of BJA-95 on CCA cells through the apoptosis pathway, especially with highly invasive KKU-213 cells. Hence, to confirm this anticancer potential in vivo, a CCA xenograft model was performed. The results revealed that BJA-95 could significantly inhibit KKU-213 cell growth in nude mice. It could also increase the expression of pro-apoptotic proteins in tumor tissues, while anti-apoptotic protein expression was decreased. However, the general toxicity to blood cells, kidneys and the liver, as well as changes in body weight, did not appear to occur. This current study was the first to demonstrate the death-inducing effect of BJA against highly invasive CCA (KKU-213) by targeting apoptosis in both in vitro and in vivo experiments. The inhibitory effect of autophagy induced by BJA-95 was to activate KKU-213 cells apoptosis. Taken together, BJA exhibited the high effectiveness against cholangiocarcinoma without discernible side effects. Moreover, therapeutic efficacy of BJA could be enhanced by combination with an autophagy inhibitor, which was a reserved strategy in the case of subsequent therapeutic resistance. Therefore, we consider BJA treatment to be an attractive therapy against bile duct cancer.

## 4. Materials and Methods

### 4.1. BJA Recipe Preparation and Extraction

The extraction process was executed according to the Thai traditional procedure that has been previously described [19]. Briefly, herbal ingredients were dried and powdered. The coarse powder of each ingredient was then mixed at different ratios according to the recipe. The BJA powder was fermented in 95% ethanol and 50% ethanol. For water extraction, the BJA powder was fermented in tamarind juice at a pH of 2.74 in a ratio of 1:4 (w:v).

### 4.2. Chemicals and Reagents

High glucose Dulbecco’s modified Eagle’s medium (DMEM-HG) (12800-58), Ham F-12 (21700-075), fetal bovine serum (FBS), phosphate-buffered saline (PBS) and trypsin-EDTA solution were purchased from Gibco (Grand Island, NY, USA). Additionally, 3-(4,5-Dimethythiazol-2-yl)-2,5-diphenyltetrazolium bromide (MTT), 3,3′-dihexyloxacarbocyanine iodide (DiOC_6_) and dimethyl sulfoxide (DMSO) were purchased from Sigma Chemical, Inc. (St. Louis, MO, USA). The substrates of caspase-9 (LEHD-para-nitroaniline; LEHD-p-NA), caspase-8 (IETD-para-nitroaniline; IETD-p-NA) and caspase-3 (DEVD-para-nitroaniline; DEVD-p-NA) were obtained from Invitrogen (Thermo Fisher Scientific Inc., (Waltham, MA, USA)). Primary antibodies against Bax (ab32503), Bid (ab2388), Puma (ab9643), Bcl-xL (ab2568), ATG-5 (ab109490), LC3B (ab58610), beta Actin (ab8227) and peroxidase-labeled secondary antibodies: anti-rabbit IgG (ab97051) and anti-mouse IgG (ab97046), were purchased from Abcam (Cambridge, UK). Primary antibodies against NF-κB p65 (6956) were purchased from Cell Signaling Technology, Inc., (Danvers, MA, USA). Primary antibodies against Beclin-1 (612113) were purchased from BD Biosciences. Annexin V Fluos staining kits and protease inhibitor cocktail tablets were obtained from Roche Diagnostics, (Mannheim, Germany). Antibodies for immunohistochemistry, anti-Bax (5023), anti-Bcl-xL (2764), were purchased from Cell Signaling Technology, Inc. and anti-active caspase 3 (559565) was purchased from BD Biosciences, (San Jose, CA, USA).

### 4.3. Cell Culture

Human cholangiocarcinoma cell lines (KKU-100, KKU-055, HuCCA-1 and KKU-213, a cell with the highest invasive and motility abilities) and immortalized human cholangiocyte MMNK-1 cell lines were obtained from the Japanese Collection of Research Bioresources (JCRB) Cell Bank, Japan. KKU-213, KKU-100, HuCCA-1 and MMNK-1 cells were cultured in Ham F-12 medium with NaHCO_3_, 100 U/mL penicillin and streptomycin. The medium was adjusted to a pH of 7.2 and supplemented with 10% heat-inactivated fetal bovine serum. KKU-055 cells were cultured in Dulbecco’s modified Eagle’s medium with 25 mM NaHCO_3_, 100 units/mL penicillin, 100 μg/mL streptomycin and supplemented with 10% heat-inactivated fetal bovine serum. All cell lines were cultured at 37°C in an incubator supplied with 5% of CO_2_.

### 4.4. Cell Viability Assay

Cell lines were seeded in 96-well culture plates for 70–80% confluence before treatment with various concentrations of BJA for 24 h of incubation. The cell viability at each concentration was determined by a 3-(4,5 dimethylthiazol-2yl)-2,5 diphenyltetrazolium bromide (MTT) assay [49]. The resulting value was then compared with that of the control or that of the untreated conditions [50]. The percentage of cell viability was used for calculating the inhibitory concentration (IC) values by using nonlinear regression (curve fitting analysis) in GraphPad Software version 6 (San Diego, CA, USA).

### 4.5. Apoptosis Assay

After cells were treated with BJA for 24 h, the culture media and cell lines were collected. Cells were then washed with phosphate-buffered saline (PBS). After that, cells were stained with annexin V-fluorescein isothiocyanate (FITC) and propidium iodide (PI) dye for 15 min of incubation and analyzed by flow cytometry (CyAn ADP, Beckman Coulter, Brea, CA, USA) [51].

### 4.6. Western Blotting Analysis

After treatment with BJA, cells were lysed and proteins were then extracted by RIPA buffer containing supplements with a protease inhibitor. Immunoblotting was performed as has been previously described [52]. Antibodies against Bax, PUMA, Bcl-xL, Bid and NF-κB p65 (1:1000 dilution), Beclin-1, ATG-5 and LC3B (1:500 dilution) and β-actin (1:10000 dilution) were employed to determine the expression levels of each protein. Protein bands were visualized using a Super Signal West Pico chemiluminescence ECL kit (Thermo Fisher Scientific Inc., Waltham, MA, USA). X-ray films were then examined for signal detection. After the developed film was scanned with an HP Scanjet G4010, the band intensities were quantified using ImageJ software [53]. The density values of each protein were normalized with the density value of β-actin.

### 4.7. Determination of Caspase Activities

The determination of caspase activity was achieved using DEVD-p-NA, IETD-p-NA and LEHD-p-NA, specific chromogenic substrates of caspase-3, caspase-8 and caspase-9, respectively. After cells were treated with BJA for 24 h, whole cell protein lysates were collected. Chromogenic substrates were then added and the specimens were incubated at 37 °C for an hour. The optical density was measured at a wavelength of 405 nm using a microplate reader (Synergy H4, BioTek, Winooski, VT, USA) [50].

### 4.8. Determination of Mitochondrial Transmembrane Potential

This method was conducted according to a previously described one [51]. Briefly, after treatment with BJA, cells were collected and were then washed with PBS before being stained with 40 nM of 3,3′-dihexyloxacarbocyanine iodide (DiOC_6_) for 15 min at 37 °C. After that, cells were analyzed by flow cytometry (CyAn ADP, Beckman Coulter, Brea, CA, USA).

### 4.9. Autophagic Vacuole Detection

Autophagic flux measurements were performed using an Autophagy Detection Kit (ab139484; Abcam, UK) according to the manufacturer’s protocol. Cells were seeded into 24-well plates at a density of 10,000 cells/well. At the end of the treatment, autophagic vacuoles were stained according to the manufacturer’s instructions before being imaged under fluorescence microscopy (ImageXpress Micro 4 High-Content Imaging System, San Jose, CA, USA) [54]. For flow cytometric analysis, after treatment with BJA, cells were collected and then washed with 1× assay buffer. After that, autophagosomes and autolysosomes were stained with a green fluorescence dye for 30 min of incubation and then analyzed quantitatively by flow cytometry [55].

### 4.10. Experimental Animals

Approximately 2 × 10^6^ cholangiocarcinoma (KKU-213) cells in 0.5 mL Ham’s F-12 medium were injected into the skin on the dorsal flank in order to reach the subcutaneous pocket of eight-week-old male athymic BALB/c nude mice (M-CLEA Bioresource Co., Ltd., Thailand). After 3 days, mice were randomized into three groups (five mice per group). Group 1 was labeled as the control mice and was fed with a mixture of distilled water and Tween-80 (4:1, *v*:*v*) 10 mL/kg. Groups 2 and 3 were identified as experimental groups. In these groups, mice were treated daily with BJA-95 at 25 mg/kg/day and 50 mg/kg/day by oral gavage for 20 days. Body weight and diameter of the tumors (length and width) were measured using a caliper ruler beginning on the initial day of grouping, and the measurements were assessed twice a week. On day 21, all mice were sacrificed and the tumor masses were removed and weighed [56]. Formalin-fixed paraffin-embedded sections of the tumor masses were analyzed by hematoxylin and eosin (H&E) staining according to the standard method. Necrosis was determined by a pathologist in a blind manner, and the total tumor mass was subtracted from the necrotic area to calculate the viable tumor mass [57]. Complete blood count and blood chemistry were analyzed by the Laboratory of Hematology and Biochemistry, Small Animal Hospital, Faculty of Veterinary Medicine, Chiang Mai University, Chiang Mai, Thailand. The in vivo study was approved by the Animal Ethics Committee, Laboratory Animal Center, Chiang Mai University, Thailand, protocol number 2562/MC-0007, the date of the approval April 24, 2019.

### 4.11. Immunohistochemistry

Immunohistochemistry was performed according to the method previously described [58]. Briefly, formalin-fixed, paraffin-embedded tissues were sectioned at a thickness of 4 µm, and tissues were then stained with anti-Bax (1:3000 dilution), anti-Bcl-xL (1:150 dilution) and anti-active caspase-3 antibody (1:100 dilution) using a Ventana automated immunostainer (Ventana Medical Systems, Tucson, AZ, USA) according to the manufacturer’s stated protocol. Quantification of immunoreactivity was assessed using ImageJ Fiji software, version 1.2 [59,60].

### 4.12. Statistical Analysis

Values are presented as mean ± standard deviation (SD) and statistical analysis was performed by statistical SPSS Software version 20. Differences between three or more groups were analyzed by one-way ANOVA multiple comparisons followed by Tukey’s test. A Student’s *t*-test was used to compare differences between the two groups. Differences in the values were considered statistically significant when the *p*-value was < 0.05.

## Figures and Tables

**Table 1:** The half maximal inhibitory concentration (IC50) of BJA on CCA cell lines.

CCA Cell Lines	IC50 (µg/mL)		
	BJA-95	BJA-50	BJA-W
KKU-213	7.1 ± 1.3	11.1 ± 1.5 ^#^	>100
HuCCA-1	17.9 ± 1.2 **	28.2 ± 3.5 ^##^	>100
KKU-100	21.0 ± 2.8 **	40.0 ± 2.5 ^###^	>100
KKU-055	23.3 ± 1.8 ***	61.7 ± 8.9 ^##^	>100

Notes: Results are shown as mean ± SD, *n* = 3. (**) *p* < 0.01, (***) *p* < 0.001 compared with BJA-95 of KKU-213 cells. (#) *p* < 0.05, (##) *p* < 0.01, (###) *p* < 0.001 compared with BJA-95 of individual cells.

**Table 2:** Complete blood count and blood chemistry of mice treated with BJA-95 compared with control.

Parameters	Control	BJA-95	
		25 mg/kg	50 mg/kg
Complete blood count (CBC)			
Red blood cells (×10^6^/µL)	8.8 ± 0.6	9.4 ± 0.6	9.2 ± 0.9
Hemoglobin (g/dL)	13.9 ± 1.0	15.1 ± 1.1	14.6 ± 1.8
Hematocrit (%)	43.8 ± 3.4	47.3 ± 3.2	46.1 ± 5.6
Mean corpuscular volume (MCV) (fL)	49.8 ± 0.6	50.2 ± 0.4	50.2 ± 1.4
Mean corpuscular hemoglobin (MCH) (pg)	15.4 ± 0.2	16.0 ± 0.3	15.9 ± 0.5
Mean corpuscular hemoglobin concentration (MCHC) (g/dL)	31.8 ± 0.6	31.9 ± 0.4	31.7 ± 0.4
White blood cells (×10^3^/µL)	11.9 ± 4.8	9.3 ± 3.8	12.1 ± 10.1
Neutrophil (%)	56.8 ± 11.7	37.3 ± 14.1	36.1 ± 21.0
Lymphocyte (%)	21.1 ± 7.4	34.8 ± 17.3	33.1 ± 16.1
Monocyte (%)	15.5 ± 5.9	25.0 ± 12.6	27.2 ± 12.4
Eosinophil (%)	4.9 ± 1.5	3.8 ± 0.8	3.4 ± 1.6
Basophil (%)	0.8 ± 1.6	0.1 ± 0.1	0.1 ± 0.2
Platelet (×10^3^/µL)	691.7 ± 323.0	915.4 ± 258.1	949.8 ± 168.8
Blood chemistry (kidney)			
Blood urea nitrogen (BUN) (mg/dL)	23.9 ± 2.4	21.0 ± 3.6	24.8 ± 4.3
Creatinine (mg/dL)	0.5 ± 0.1	0.5 ± 0.1	0.4 ± 0.1
Blood chemistry (liver)			
Aspartate aminotransferase (AST) (U/L)	102.2 ± 20.8	113.8 ± 49.1	94.0 ± 28.5
Alanine aminotransferase (ALT) (U/L)	107.2 ± 93.8	70.4 ± 82.6	67.0 ± 18.3

Notes: Results are shown as mean ± SD, (*n* = 5).

## Supplementary Materials

Supplementary materials can be found at https://www.mdpi.com/1422-0067/21/16/5669/s1.

## Abbreviations

ALT	alanine aminotransferase
Annexin V-FITC/PI	annexin V-fluorescein isothiocyanate and propidium iodide
AST	aspartate aminotransferase
BJA	Benja Amarit
BJA-W	water extract of BJA
BJA-50	50% ethanolic extract of BJA
BJA-95	95% ethanolic extract of BJA
BUN	blood urea nitrogen
CCA	cholangiocarcinoma
DEVD-p-NA	Asp-Glu-Val-Asp para-nitro aniline
DiOC_6_	3,3′-dihexyloxacarbocyanine iodide
ER	endoplasmic reticulum
HCC	hepatocellular carcinoma
H&E	hematoxylin and eosin
IETD-p-NA	Ile-Glu-Thr-Asp para-nitro aniline
LEHD-p-NA	Leu-Glu-His-Asp para-nitro aniline
MCH	mean corpuscular hemoglobin
MCHC	mean corpuscular hemoglobin concentration
MCV	mean corpuscular volume
NF-κB	nuclear factor kappa light chain enhancer of activated B cells
3-MA	3-methyladenine
