# Supplementary material for: Antitumor Efficacy of the Herbal Recipe Benja Amarit against Highly Invasive Cholangiocarcinoma by Inducing Apoptosis both In Vitro and In Vivo
Source: Int J Mol Sci. 2020 Aug 7;21(16):5669. doi: 10.3390/ijms21165669 (PMC7460969; doi:10.3390/ijms21165669)
Supplement: Supplementary file 1 [file ijms-21-05669-s001.pdf]

Supplementary Information

Table S1. The half maximal inhibitory concentration (IC_{50}) and 50% hemolysis (HC_{50}) of BJA-95 on normal cells compared with KKU-213 cell.

**Cell type**	**Cell lines**	**IC_{50} (μg/ml)**
Cholangiocarcinoma cell	KKU-213	7.1±1.3
Normal cholangiocyte	MMNK-1	10.5±1.6*
Human breast epithelial cell	MCF-10A	16.1±1.4**
Fibroblast cell	3T3-L1	11.6±1.1*
Blood cell	Peripheral blood mononuclear cells (PBMCs)	13.8±1.7**
		**HC_{50} (mg/ml)**
Blood cell	Red blood cells (RBCs)	0.5±0.1****

Notes: Results are shown as mean ± SD from repeated three independent experiments for KKU-213 and MMNK-1 cell lines. The IC_{50} and HC_{50} on MCF-10A, 3T3-L1, PBMCs and RBCs were referred from our previous study [19]. (*) $p<0.05$, (**) $p<0.01$, (***) $p<0.001$, (****) $p<0.0001$ significantly higher than the value of KKU-213 cells.

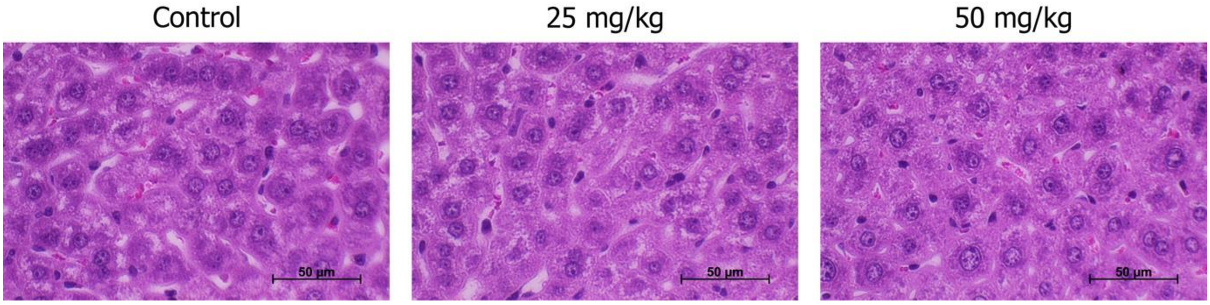

Figure S1. Representative photos of hematoxylin and eosin (H&E) staining of liver tissues. The BJA-95 treatment showed no discernible toxicity on mice liver cells.
